# The Fused Methionine Sulfoxide Reductase MsrAB Promotes Oxidative Stress Defense and Bacterial Virulence in Fusobacterium nucleatum

**DOI:** 10.1128/mbio.03022-21

**Published:** 2022-04-14

**Authors:** Matthew Scheible, Cuong T. Nguyen, Truc Thanh Luong, Ju Huck Lee, Yi-Wei Chen, Chungyu Chang, Manuel Wittchen, Martha I. Camacho, Bethany L. Tiner, Chenggang Wu, Andreas Tauch, Asis Das, Hung Ton-That

**Affiliations:** a Division of Oral Biology and Medicine, School of Dentistry, University of California, Los Angeles, California, USA; b Department of Microbiology & Molecular Genetics, University of Texas McGovern Medical School, Houston, Texas, USA; c Korean Collection for Type Cultures, Korea Research Institute of Bioscience and Biotechnology, Daejeon, Republic of Korea; d Center for Biotechnology (CeBiTec), Bielefeld University, Bielefeld, Germany; e Department of Molecular Biology and Biophysics, University of Connecticut Health Center, Farmington, Connecticut, USA; f Molecular Biology Institute, University of California, Los Angeles, Los Angeles, California, USA; KUMC

**Keywords:** *Fusobacterium nucleatum*, MsrAB, adherence, cell invasion, gene regulation, oxidative stress, preterm birth, virulence

## Abstract

Fusobacterium nucleatum, an anaerobic Gram-negative bacterium frequently found in the human oral cavity and some extra-oral sites, is implicated in several important diseases: periodontitis, adverse pregnancy outcomes, and colorectal cancer. To date, how this obligate anaerobe copes with oxidative stress and host immunity within multiple human tissues remains unknown. Here, we uncovered a critical role in this process of a multigene locus encoding a single, fused methionine sulfoxide reductase (MsrAB), a two-component signal transduction system (ModRS), and thioredoxin (Trx)- and cytochrome *c* (CcdA)-like proteins, which are induced when fusobacterial cells are exposed to hydrogen peroxide. Comparative transcriptome analysis revealed that the response regulator ModR regulates a large regulon that includes *trx*, *ccdA*, and many metabolic genes. Significantly, specific mutants of the *msrAB* locus, including *msrAB*, are sensitive to reactive oxygen species and defective in adherence/invasion of colorectal epithelial cells. Strikingly, the *msrAB* mutant is also defective in survival in macrophages, and it is severely attenuated in virulence in a mouse model of preterm birth, consistent with its failure to spread to the amniotic fluid and colonize the placenta. Clearly, the MsrAB system regulated by the two-component system ModRS represents a major oxidative stress defense pathway that protects fusobacteria against oxidative damage in immune cells and confers virulence by enabling attachment and invasion of multiple target tissues.

## INTRODUCTION

The Gram-negative bacterium Fusobacterium nucleatum is associated with a number of clinically important human conditions. An obligate anaerobe often found in the dental plaque (1), fusobacteria occupy the intermediate annulus within the oral microbial biofilms that hold together the base and peripheral layers, each with distinct microbial compositions (2). Classic microbial studies have uncovered a key role of F. nucleatum in polymicrobial interactions, central to the formation of multispecies oral biofilms (3). Although generally regarded as a commensal (4, 5), F. nucleatum has the remarkable ability to spread to distal extraoral sites, where it is associated with a number of diseases, including adverse pregnancy outcomes, colorectal cancer, and breast cancer (6–10). F. nucleatum has turned up as one of the most prevalent species in adverse pregnancy outcomes (4), and it has been detected in various placental and fetal tissues as well as amniotic fluid, cord blood, and fetal organs (6, 11–14). DNA lineage analysis has confirmed that F. nucleatum can spread from the subgingival plaque to the placenta and fetus, leading to pregnancy complications, including inflammation and stillbirth (13, 15, 16). Congruent with this, Han and colleagues provided the first experimental evidence that hematogenous spread by F. nucleatum causes placental colonization and preterm birth in a murine model (17). While the pathophysiological mechanisms of preterm birth induced by F. nucleatum are currently unknown, a few adhesins have been suggested to play a role in placental colonization (4). For example, initially identified as an adhesin involved in the attachment and invasion of epithelial cells (18), FadA promotes fusobacterial colonization of the mouse placenta (19). Another identified factor, the outer membrane protein Fap2, is a multifunctional protein; Fap2 induces cell death in human lymphocytes (20), binds to host d-galactose-β(1–3)-*N*-acetyl-d-galactosamine (Gal-GalNAc), which is highly expressed in colorectal cancer cells (21), and is involved in murine placental colonization (22). Importantly, no fusobacterial factors studied to date have been shown to be critically required for induction of preterm birth and defense against oxidative stress imposed by placental immune cells, such as infiltrating neutrophils and placental macrophages.

Activated neutrophils and macrophages control infection by extracellular or intracellular pathogens by producing phagosomal cytotoxic reactive oxygen species, including cytotoxic superoxide radicals and hydrogen peroxide (23). These reactive species damage proteins by oxidation of the methionine residues. As part of a key defensive mechanism, both eukaryotic and prokaryotic cells produce methionine sulfoxide reductases, which repair oxidized methionine residues by reducing them back to methionine (24). A methionine sulfoxide reductase, termed MsrA, was purified from E. coli, which selectively reduces the S-form of methionine sulfoxide (25). Subsequently, a second methionine sulfoxide reductase (MsrB) was identified and shown to reduce the R-form of methionine sulfoxide (26). Most methionine sulfoxide reductases are encoded as individual enzymes (27). Certain bacteria, including Streptococcus pneumoniae, Helicobacter pylori, and Treponema denticola, however, encode these two reductases as a single fusion protein (MsrAB) (27–30). Intriguingly, in some bacterial species, including S. pneumoniae and Streptococcus gordonii, *msrAB* are genetically linked to genes coding for thioredoxin (Trx) and cytochrome *c*-type proteins (CcdA) (31, 32), which participate in the regeneration of MsrAB reductase activity (29, 32, 33). Given their function in repairing oxidized proteins, including virulence factors, the role of MsrA/B in bacterial pathogenesis has been examined (24). Indeed, a S. pneumoniae mutant devoid of *msrAB* exhibits not only reduced survival in macrophages but also decreased virulence and dissemination in a mouse model of infection (29). Likewise, in S. gordonii, MsrAB is required for oxidative stress tolerance, biofilm formation, and oral colonization in mice (32); S. gordonii encodes a second methionine sulfoxide reductase called MsrA, which also plays a role in oxidative stress defense and bacterial adherence (34).

In the present work, we investigated the presumptive roles of methionine sulfoxide reductases in oxidative stress defense and virulence in the obligate anaerobe F. nucleatum to provide new knowledge of key pathogenic mechanisms. Here, we report the identification and functional characterization of the fused methionine sulfoxide reductase MsrAB in F. nucleatum. Intriguingly, the fusobacterial MsrAB is part of a five-gene locus that encodes a two-component transduction system (TCS) ModRS, Trx- and CcdA-like proteins. Transcriptome analysis by transcriptome sequencing (RNA-seq) revealed that this TCS modulates expression of a large regulon including *msrAB*, *trx*, and *ccdA*. Significantly, various mutants lacking individual genes in this locus are sensitive to hydrogen peroxide and also are defective in adherence/invasion and survival in macrophages. We demonstrate further that the *msrAB* mutant fails to induce preterm birth in a mouse model. Unlike the parent strain, the *msrAB* mutant is unable to colonize the mouse placenta, reducing spread to different organs upon infection.

## RESULTS

### Oxidative stress response in fusobacteria: stress induction of a gene locus encoding a fused methionine sulfoxide reductase.

**FIG 1:**
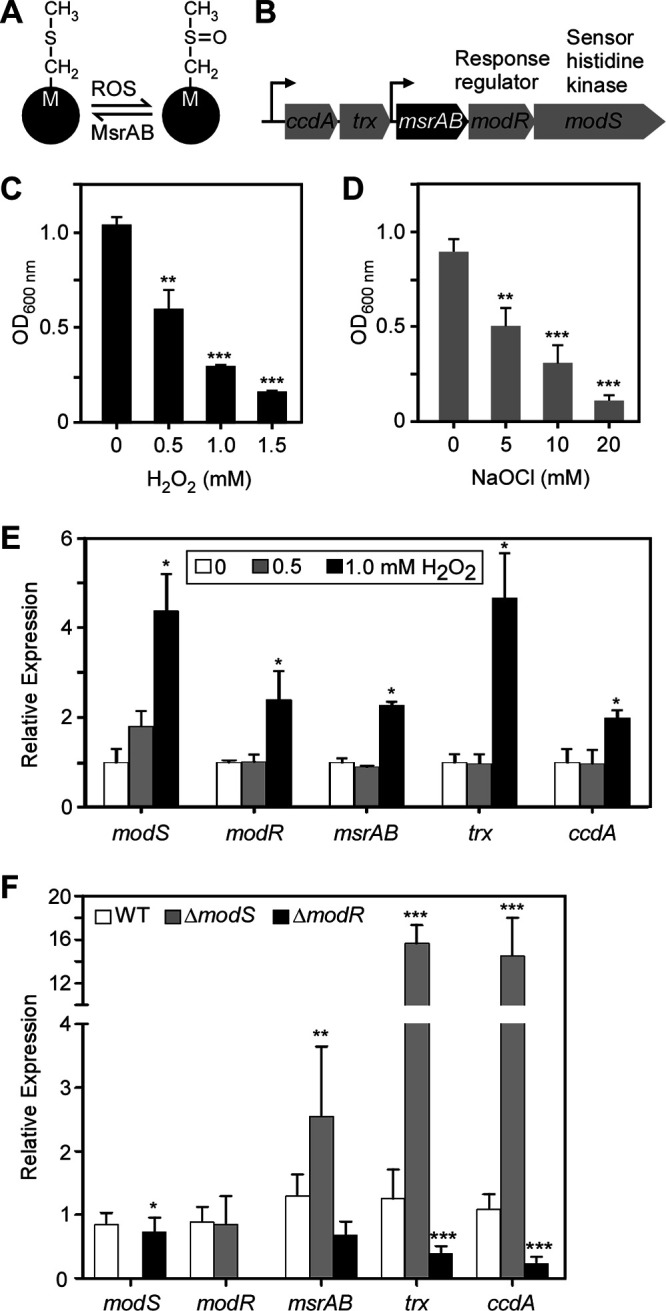
Oxidative stress response by the *msrAB* gene locus in F. nucleatum. (A) Methionine (M) in proteins (black circles) is known to be sensitive to oxidation by reactive oxygen species (ROS), which converts methionine to methionine sulfoxide. Methionine sulfoxide reductase enzymes MsrAB catalyze reduction of methionine sulfoxide to methionine. (B) F. nucleatum harbors an *msrAB* locus, which encodes thioredoxin (Trx)- and CcdA-like proteins, methionine sulfoxide reductase MsrAB, a response regulator (ModR), and a sensor histidine kinase (ModS). (C and D) Log-phase cultures of the fusobacterial parental strain were treated with increasing concentrations of H_2_O_2_ (C) or NaOCl (D). Cell growth was determined by optical density at 600 nm (OD_600_) after 24 h of treatment. The results are presented as averages from three independent experiments performed in triplicate. (E) Log-phase cultures of the fusobacterial parental strain were mock treated or treated with 0.5 or 1.0 mM H_2_O_2_ for 8 h. Expression of indicated genes was determined by quantitative reverse transcription-PCR (qRT-PCR). Relative expression of each gene is presented as an average from two independent experiments performed in triplicate with 16S mRNA used as a control. (F) The parental strain and its Δ*modS* and Δ*modR* isogenic mutants were treated with 1 mM H_2_O_2_ for 8 h. Gene expression was determined by qRT-PCR as described for panel E. Statistical analysis was performed with GraphPad (*, *P* < 0.05; **, *P* < 0.01; ***, *P* < 0.001).

As previously mentioned, reactive oxygen species (ROS) oxidize methionine residues in proteins to methionine sulfoxides, which are enzymatically converted back to methionine by MsrA and MsrB (Fig. 1A). Using BLAST and homology searches, we found that the genome of F. nucleatum ATCC 23726 contains a locus predicted to encode a single, fused methionine sulfoxide reductase, which we named MsrAB (Fig. 1A and B). Intriguingly, upstream of *msrAB*, there are two genes that are predicted to encode thioredoxin (Trx)- and cytochrome *c* (CcdA)-like proteins. Two genes are located downstream of *msrAB* and encode a two-component signal transduction system (TCS), which we named ModRS (Mod stands for mediator of oxidative stress defense) (Fig. 1B).

To first examine if the *msrAB* gene locus is responsive to ROS treatment, we determined the levels of ROS exposure that F. nucleatum is able to withstand, using H_2_O_2_ or NaOCl as sources of ROS. Fusobacterial cells were sensitive to all tested concentrations of H_2_O_2_ (Fig. 1C) and NaOCl (Fig. 1D), with increasing dose-dependent inhibition. We next determined whether the expression of the *msrAB* locus was altered by treating fusobacterial cells with lower concentrations (0.5 or 1 mM) of H_2_O_2_ for 8 h. As determined by quantitative reverse transcription-PCR (qRT-PCR), only *modS* was slightly upregulated at 0.5 mM H_2_O_2_ (Fig. 1E), while increasing the concentration of H_2_O_2_ to 1 mM significantly upregulated all the genes of the *msrAB* locus (Fig. 1E), suggesting that a minimal threshold of H_2_O_2_ (and, hence, oxidative damage) must be reached to cause an optimal induction of the locus.

Next, to test whether ROS triggers a more widespread change in gene expression, we performed comparative transcriptomic analysis employing RNA sequencing (RNA-seq) technology (see Materials and Methods). Using a 2-fold cutoff (log_2_ fold change, ±1), we found 145 genes to increase in expression with H_2_O_2_ treatment, while 86 genes were downregulated (see Table S1 in the supplemental material). Significantly, among the genes that showed significantly increased expression are those encoding proteins predicted to be involved in metabolism, transport, and transcriptional regulation. Notable classes of genes whose expression was decreased or repressed are those predicted to encode nucleotide biosynthesis proteins, oxidases, some transporters, and transcriptional regulators. Intriguingly, genes downregulated by oxidative stress included several known adhesin genes, *fadA*, *fap2*, and *radD*. Together, these data reveal that oxidative stress induced by H_2_O_2_ has a significant impact on global gene expression, leading to the up- or downregulation of genes that are involved in detoxification, nucleotide biosynthesis, transport and transcriptional regulation, and adhesion and invasion, as may be expected for the survival of a successful bacterial pathogen.

10.1128/mbio.03022-21.4 TABLE S1 Differentially expressed genes in the Δ*modR* mutant relative to the untreated parental strain. Download Table S1, XLSX file, 0.1 MB. Copyright © 2022 Scheible et al. 2022 Scheible et al. https://creativecommons.org/licenses/by/4.0/ This content is distributed under the terms of the Creative Commons Attribution 4.0 International license.

### ModR serves as a critical oxidative stress response regulator for a large number of stress-sensitive genes in fusobacteria.

Since the TCS-encoding genes *modS* and *modR* are linked to *msrAB* in a gene cluster and also induced by oxidative stress, it was important to determine whether and how the TCS ModSR affects expression of the *msrAB* gene locus by qRT-PCR analysis. Deletion of the *modS* gene resulted in a 3-fold increase in *msrAB* expression and more than 15-fold increase in *trx* and *ccdA* expression (Fig. 1F). In contrast, *modR* deletion significantly decreased expression of *trx* and *ccdA*, accompanied by a slight decrease in *modS* expression (Fig. 1F). Therefore, the response regulator ModR acts as an activator of *trx* and *ccdA* while the sensor kinase ModS inhibits transcription of these same genes, presumably by inactivation of ModR via phosphorylation at the conserved residue D55. To examine if mutations of this residue affect expression of *msrAB*, *trx,* and *ccdA*, we generated D55A and D55E mutations on ModR, and recombinant plasmids expressing wild-type ModR or ModR mutants were introduced into the Δ*modR* mutant. Expression of these genes in mutant strains was then compared with that of the parent strain by qRT-PCR. Compared to the Δ*modR* mutant, which reduced expression of *msrAB*, *trx,* and *ccdA*, the strain expressing ModR (Δ*modR*/pModR) increased expression of the three genes (Fig. S1). In contrast, the ModR_D55A_ mutant produced the same level of gene expression as the Δ*modR* mutant, whereas the ModR_D55E_ mutant mirrored the Δ*modR*/pModR strain (Fig. S1). This is consistent with the predicted role of ModS in inactivation of ModR by phosphorylation.

10.1128/mbio.03022-21.1 FIG S1 Effects on gene expression by mutations of the conserved D55 residue of ModR. Total RNA samples extracted from the parent, Δ*modR* mutant, and its derivative strains grown to mid-log phase were used to generate cDNA for quantification of gene expression by qRT-PCR, with probes targeting *msrAB*, *trx*, and *ccdA*. Expression of *msrAB*, *trx*, and *ccdA* in the Δ*modR* mutant and its derivatives are presented as fold change, relative to that of the parent strain, from experiments performed in triplicate in three biological repeats and analyzed by GraphPad (***, *P* < 0.001). Download FIG S1, PDF file, 0.1 MB. Copyright © 2022 Scheible et al. 2022 Scheible et al. https://creativecommons.org/licenses/by/4.0/ This content is distributed under the terms of the Creative Commons Attribution 4.0 International license.

Next, we determined whether ModR-mediated activation of *trx* and *ccdA* is limited to these two genes or whether ModR acts a global regulator of other stress response genes unlinked to the ModRS locus. Using RNA-seq we compared the whole transcriptomes of the wild-type F. nucleatum strain and its Δ*modR* mutant. Using a 2-fold cutoff (log_2_ fold change, ±1) to probe differential gene expression under this condition, we uncovered a set of 177 genes whose expression was elevated in the Δ*modR* mutant, while 147 genes showed diminished expression (Table S1). Differential expression of randomly selected genes was also confirmed by qRT-PCR (Fig. S2). Intriguingly, the majority of the upregulated genes (i.e., those that are repressed directly or indirectly by ModR) are predicted to be involved in metabolism, with genes coding for ethanolamine utilization being very highly expressed (for example, 45-fold increase observed for *eutM*; Table 1). In contrast, the expression of genes coding for factors involved in glycolysis and nucleotide biosynthesis were most highly diminished, meaning that their expression is normally activated by ModR (Table 2). Encoding a galactose-inhibitable adhesin that is required for polymicrobial interactions and that promotes fusobacterial enrichment in colorectal cancer tissues (21, 22), *fap2* mRNA is significantly reduced in the Δ*modR* mutant (Table 2 and Fig. S2). ModR acts, therefore, as an activator of a key adhesin and virulence factor and has a broad role in the differential regulation of a large regulon that includes activated genes such as *trx*, *ccdA,* and *fap2* and repressed genes such as *eutM* and others involved in ethanolamine utilization.

**TABLE 1:** Unregulated genes in the Δ*modR* mutant relative to the parental strain

Locus ID and category	Gene	Predicted function	Fold change^*a*^
Metabolism			
HMPREF0397_RS01515	*eutM*	Ethanolamine utilization microcompartment protein	45.77
HMPREF0397_RS01520		Acetaldehyde dehydrogenase (acetylating)	43.11
HMPREF0397_RS01530		Ethanolamine utilization protein	42.28
HMPREF0397_RS01525		Ethanolamine utilization protein	40.01
HMPREF0397_RS01505	*eutL*	Ethanolamine utilization microcompartment protein	36.57
HMPREF0397_RS01510		BMC domain-containing protein	34.58
HMPREF0397_RS01545	*eutH*	Ethanolamine utilization protein	33.96
HMPREF0397_RS01500		Ethanolamine ammonia-lyase subunit	32.80
HMPREF0397_RS01535		Ethanolamine utilization protein	30.67
HMPREF0397_RS04485	*megL*	Methionine gamma-lyase	15.27
HMPREF0397_RS01490	*eutA*	Ethanolamine ammonia-lyase reactivating factor	14.06
HMPREF0397_RS04495	*nifJ*	Pyruvate-ferredoxin (flavodoxin) oxidoreductase	12.56
HMPREF0397_RS01550		DUF861 domain-containing protein	9.35
HMPREF0397_RS06445		NAD(P)/FAD-dependent oxidoreductase	7.08
HMPREF0397_RS03635		Urocanate hydratase	6.97
HMPREF0397_RS03630	*hutH*	Histidine ammonia-lyase	6.57
HMPREF0397_RS02330		Acyl-CoA^*b*^ dehydrogenase	6.57
HMPREF0397_RS02335		Electron transfer flavoprotein subunit beta	6.27
HMPREF0397_RS02340		Electron transfer flavoprotein subunit alpha	6.04
HMPREF0397_RS04970		Formate-tetrahydrofolate ligase	5.85
HMPREF0397_RS07365	*ftcD*	Glutamate formimidoyltransferase	5.27
HMPREF0397_RS02930		Glu/Leu/Phe/Val dehydrogenase	5.16
HMPREF0397_RS07355		Formiminotetrahydrofolate cyclodeaminase	4.46
HMPREF0397_RS04270		Tyrosine phenol-lyase	4.35
HMPREF0397_RS07360		Imidazolonepropionase	4.27
HMPREF0397_RS05360		CoA transferase subunit B	4.07
Redox			
HMPREF0397_RS06440		FAD-dependent oxidoreductase	20.93
HMPREF0397_RS02280		Ferredoxin family protein	4.10
Transport			
HMPREF0397_RS04265		Sodium-dependent transporter	4.56
HMPREF0397_RS04490		Na^+^/H^+^ antiporter NhaC family protein	13.99
Miscellaneous			
HMPREF0397_RS05020		Prepilin peptidase	14.31
HMPREF0397_RS04990		Type II secretion system protein GspD	5.88
HMPREF0397_RS08325		Toxin-antitoxin system YwqK family antitoxin	5.81
HMPREF0397_RS02345		CidA/LrgA family protein	5.41
HMPREF0397_RS02350		LrgB family protein	5.25
HMPREF0397_RS08425		Carbon starvation protein A	5.13
HMPREF0397_RS02835		Iron-containing alcohol dehydrogenase	4.91
Hypothetical			
HMPREF0397_RS01540		Hypothetical protein	45.46
HMPREF0397_RS06435		DUF1667 domain-containing protein	27.91
HMPREF0397_RS10920		Hypothetical protein	6.06
HMPREF0397_RS04995		Hypothetical protein	5.19
HMPREF0397_RS01555		Hypothetical protein	4.83

a Selected are genes with a 4-fold cutoff (log_2_ fold change ± 2).

b CoA, coenzyme A.

**TABLE 2:** Downregulated genes in the Δ*modR* mutant relative to the parental strain

Locus ID and category	Gene	Predicted function	Fold change^*a*^
Metabolism			
HMPREF0397_RS07865	*pfkB*	1-Phosphofructokinase	−65.76
HMPREF0397_RS07860		PTS transporter subunit EIIA	−11.99
HMPREF0397_RS09965		Phosphoribosylglycinamide formyltransferase	−10.19
HMPREF0397_RS09940		Phosphoribosylformylglycinamidine synthase	−10.12
HMPREF0397_RS09945	*purE*	5-(Carboxyamino)imidazole ribonucleotide mutase	−9.84
HMPREF0397_RS09960		Phosphoribosylformylglycinamidine cyclo-ligase	−9.28
HMPREF0397_RS09955		Amidophosphoribosyltransferase	−8.51
HMPREF0397_RS09950		Phosphoribosylaminoimidazolesuccinocarboxamide synthase	−8.34
HMPREF0397_RS09980	*purH*	Bifunctional phosphoribosylaminoimidazolecarboxamide formyltransferase/IMP cyclohydrolase	−8.27
HMPREF0397_RS09985	*purD*	Phosphoribosylamine-glycine ligase	−7.39
HMPREF0397_RS04385		Glutaminase A	−5.63
HMPREF0397_RS05685		Bifunctional glycosyltransferase family 2	−5.22
Miscellaneous			
HMPREF0397_RS07870		DeoR/GlpR transcriptional regulator	−49.32
HMPREF0397_RS09970		Class I SAM-dependent methyltransferase	−10.12
HMPREF0397_RS02460		Inositol phosphorylceramide synthase	−4.75
HMPREF0397_RS07830	*fap2*	Galactose-inhibitable adhesin Fap2	−4.48
HMPREF0397_RS05955		Serine dehydratase subunit alpha family protein	−4.07
Hypothetical			
HMPREF0397_RS10740		Hypothetical protein	−64.73
HMPREF0397_RS09975		Hypothetical protein	−8.84
HMPREF0397_RS02700		Hypothetical protein	−4.17

a Selected are genes with a 4-fold cutoff (log_2_ fold change, ±2).

10.1128/mbio.03022-21.2 FIG S2 Confirmation of gene expression in the Δ*modR* mutant by qRT-PCR. Total RNA samples extracted from the Δ*modR* and parent strains grown to mid-log phase were used to generate cDNA for quantification of gene expression by qRT-PCR, with probes targeting randomly selected genes and *rpoD* as an internal control. Expression of these genes in the Δ*modR* mutant are presented as fold change relative to that of the parent strain as described for Fig. S1. All experiments were performed in triplicate in three biological repeats. Statistical analysis was performed with GraphPad (***, *P* < 0.001; n.s., not significant). Download FIG S2, PDF file, 0.1 MB. Copyright © 2022 Scheible et al. 2022 Scheible et al. https://creativecommons.org/licenses/by/4.0/ This content is distributed under the terms of the Creative Commons Attribution 4.0 International license.

**FIG 2:**
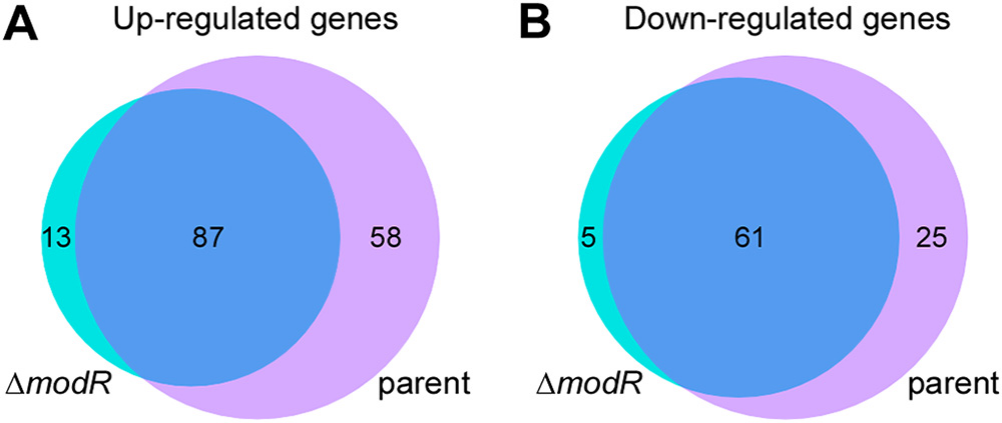
Comparative transcriptional analysis of the parental and *modR* mutant strains. RNA samples of the parental and *modR* mutant strains mock treated or treated with 1 mM H_2_O_2_ for 8 h were obtained and subjected to RNA-seq. Using a 2-fold cutoff (log_2_ fold change, ±1), upregulated (A) and downregulated (B) genes in the H_2_O_2_-treated *modR* and H_2_O_2_-treated parental strains were analyzed against the untreated parental strain.

We next compared genes that are responsive to H_2_O_2_ treatment in the presence or absence of *modR* (Fig. 2 and Tables S1 and 2). Genes were grouped based on their stress induction or stress repression, their dependence on or independence of ModR, and whether they are activated or repressed by ModR. A total of 145 genes were upregulated by oxidative stress in both parent and Δ*modR* strains, but only 58 genes were upregulated selectively in the parent strain (Fig. 2A). Since such upregulation of these genes was not evident in the absence of ModR, these genes represent ModR-responsive genes that are both ModR activated and induced by oxidative stress. By comparison, 87 genes were upregulated by stress in both the parent and the Δ*modR* mutant. Therefore, a large set of fusobacterial genes of fusobacteria is turned on by oxidative stress in a ModR-independent manner. A third class of 13 genes was stress induced only when ModR was absent. Evidently, these represent ModR-repressed stress-induced genes.

A similar observation was made for genes whose expression is diminished by oxidative stress (Fig. 2B and Table S2). Of a total of 91 such genes, we found that 61 genes were common in both Δ*modR* and parental strains in the presence of H_2_O_2_. These 61 genes are evidently depressed by oxidative stress independent of ModR. When ModR was absent, only five genes were inhibited by oxidative stress, suggesting that ModR normally represses this small set of stress-responsive genes. In contrast, 25 genes showed diminished expression upon oxidative stress only in the presence of ModR; these represent the ModR-activated genes that are downregulated by stress. We conclude that oxidative stress alters expression of a large set of fusobacterial genes in a ModR-responsive manner, while a similar number of stress-regulated genes depend on some other mechanisms for their positive and negative regulation by stress.

10.1128/mbio.03022-21.5 TABLE S2 Common up- and downregulated genes found in the Δ*modR* mutant and parental strains exposed to hydrogen peroxide relative to the untreated parental strain. Download Table S2, XLSX file, 0.02 MB. Copyright © 2022 Scheible et al. 2022 Scheible et al. https://creativecommons.org/licenses/by/4.0/ This content is distributed under the terms of the Creative Commons Attribution 4.0 International license.

### The *msrAB* locus is required for bacterial resistance to oxidative stress and efficient infection of colorectal cancer cells.

**FIG 3:**
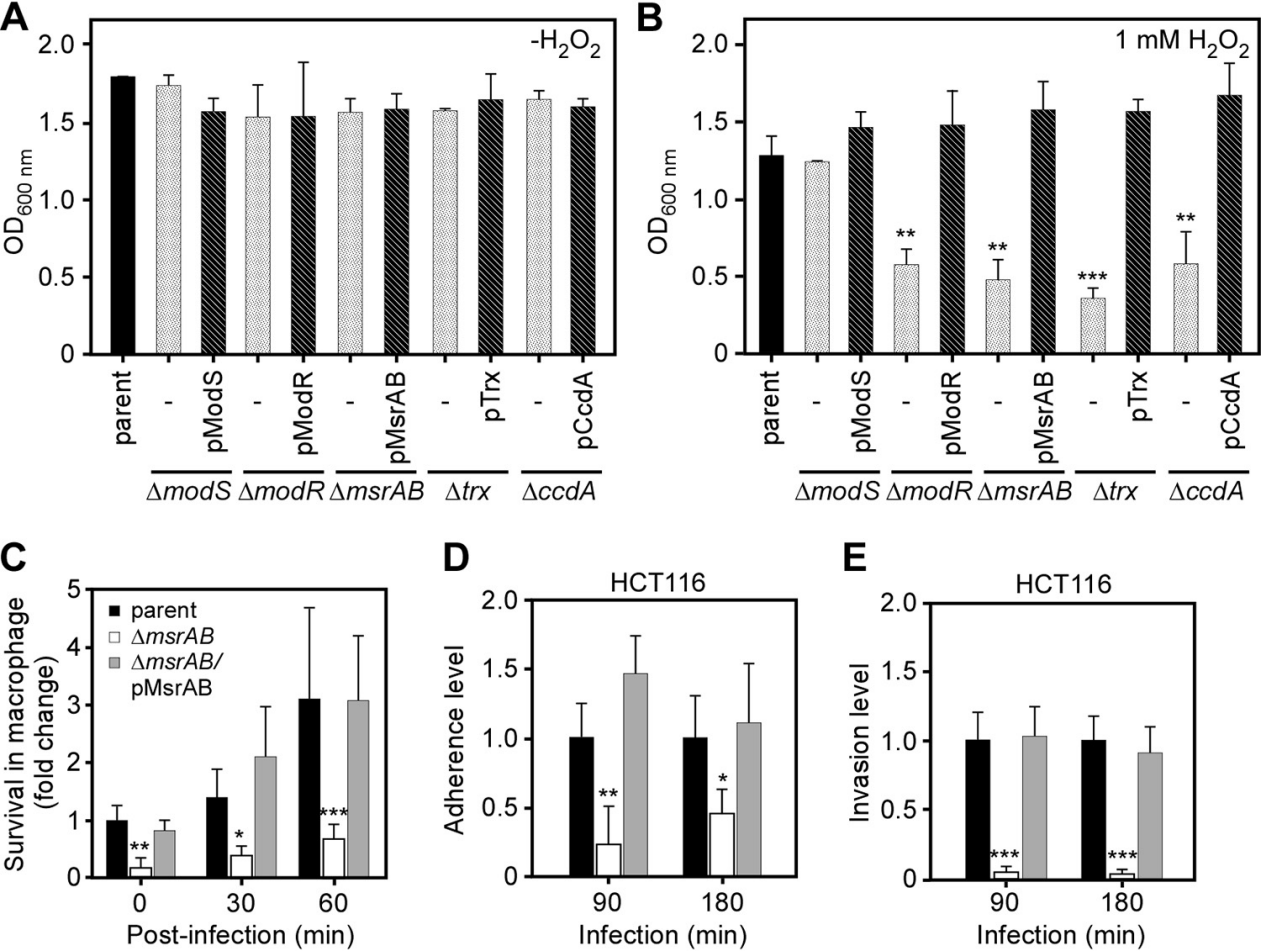
Requirement of genetic elements in the *msrAB* locus for oxidative stress resistance. (A and B) Log-phase cultures of indicated strains were mock treated (A) or treated with 1 mM H_2_O_2_ (B). Cell growth was determined by OD_600_ after 24 h of treatment. (C) RAW 264.7 macrophages were treated with the F. nucleatum parental strain, its isogenic mutant lacking *msrAB*, or this rescued strain a multiplicity of infection (MOI) of 50 for 1 h, and bacterial survival in macrophages was determined after 0, 30, or 60 min of phagocytosis. The results are presented as fold change compared with the parental strain normalized to 1. (D and E) HCT 116 colorectal cancer cells were infected with the F. nucleatum parental strain, its isogenic mutant lacking *msrAB*, or this rescued strain at an MOI of 100. Bacterial adherence (D) or invasion (E) was assessed 90 or 180 min of postinfection. Bacterial CFU were enumerated by plating on plates. Adherence and invasion efficiencies in the mutant and rescued strain were compared to the parental strain, which was normalized to 1. All values in panels A to E are averages plus standard deviations (error bars) from at least three independent experiments performed in triplicate (*, *P* < 0.05; **, *P* < 0.01; ***, *P* < 0.001).

We next asked whether the stress-induced *msrAB* locus is functionally important for stress adaptation by fusobacteria. To examine if ModR and its associated factors within the *msrAB* locus are required for bacterial adaptive response to oxidative stress and survival, we generated additional deletion mutants of fusobacteria lacking *msrAB*, *trx*, or *ccdA*. These strains showed similar cell growth in the absence of hydrogen peroxide, as expected (Fig. 3A). In the presence of H_2_O_2_, deletion of *modS* did not alter cell growth (Fig. 3B), whereas deletion of *modR*, *msrAB*, *trx*, or *ccdA* significantly reduced cell growth, and this growth defect was rescued by ectopic expression of these respective genes (Fig. 3B). Ectopic expression of these genes rescued the growth defect of the respective strains (Fig. 3B). The stress response genes with the *msrAB* locus, therefore, are each involved in protecting against damage inflicted by ROS and oxidative stress and maintaining bacterial survival.

### Importance of MsrAB in fusobacterial survival in macrophages.

Since macrophages produce ROS during phagocytic killing (35), we investigated how the *msrAB* mutant copes with engulfment by RAW 264.7 macrophages in culture. Deletion of *msrAB* significantly reduced bacterial survival and multiplication inside macrophages over time (Fig. 3C). Ectopic expression of MsrAB from a plasmid rescued the defect, demonstrating the direct role of MsrAB in the survival of bacteria in macrophages.

To examine the function of MsrAB in fusobacterial attachment and invasion of epithelial cells, we tested colorectal cancer (CRC) cells (HCT116) in standard adherence and invasion assays (see Materials and Methods). Compared to the parental bacteria, the Δ*msrAB* mutant showed a highly significant reduction in both adherence and invasion of CRC cells, which were rescued by ectopic expression of *msrAB* (Fig. 3D and E).

### Importance of multiple stress response factors in fusobacterial survival in colorectal cancer cells.

**FIG 4:**
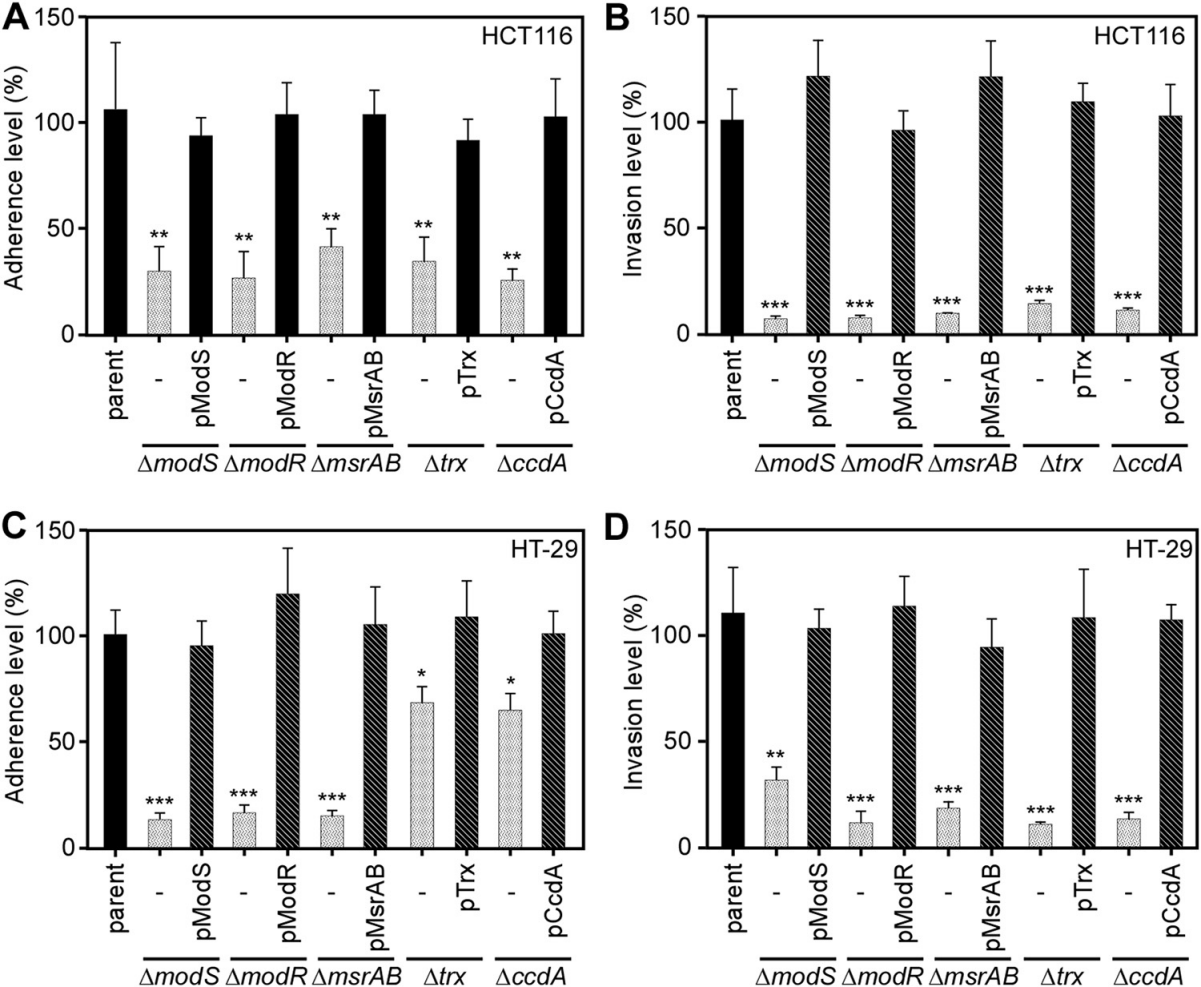
Requirement of genetic elements in the *msrAB* locus for attachment and invasion of host cells. The indicated strains were subjected to the adhesion (A and C) and invasion (B and D) assays with HCT 116 (A and B) and HT-29 (C and D) epithelial cells at an MOI of 100 as described for Fig. 3D and E. Adherence and invasion levels are presented as percentages, with all values expressed as averages plus standard deviations (error bars) from at least three independent experiments performed in triplicate (*, *P* < 0.05; **, *P* < 0.01; ***, *P* < 0.001).

To assess the role of each gene in the *msrAB* locus, we compared the parental strain, each deletion mutant, and the respective complemented strains in assays for adherence and invasion. Deletion of *modS*, *modR*, *trx*, *ccdA*, or *msrAB* caused similar reductions in adhesion and invasion of CRC HCT116 cells compared to the high level of adherence/invasion by the parental strain (Fig. 4A and B). Since these genetic defects were rescued by expression of the corresponding genes in respective mutants, the gene products appear to play direct roles in fusobacterial infection of the CRC cells. To determine whether these phenotypes were specific to CRC HCT116, we tested the strains with CRC HT-29 cells. Except for the *trx* and *ccdA* mutants, which displayed significant small reductions in adherence, the other mutants exhibited markedly reduced adherence to HT-29 cells (Fig. 4C). Remarkably, however, each of the mutant strains showed significantly greater defects in adherence-independent invasion than the parent strain (Fig. 4D). Altogether, these results indicate that MsrAB and associated factors in the *msrAB* locus promote bacterial resistance to oxidative stress and efficient infection of CRC cells and that Trx and CcdA are functionally connected to the fused methionine sulfoxide reductase MsrAB.

### MsrAB and Trx/CcdA are required for virulence in an experimental model of preterm birth.

**FIG 5:**
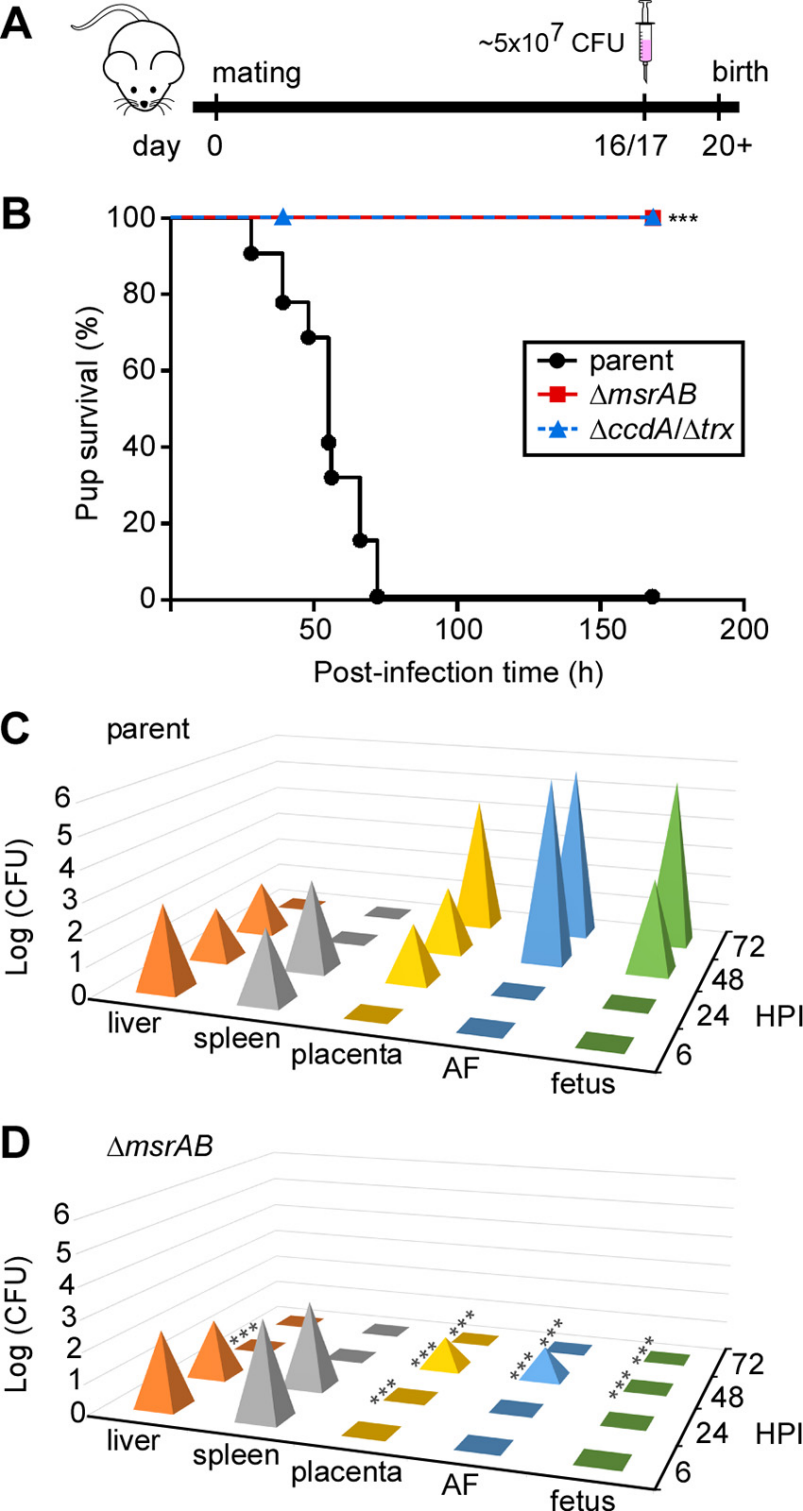
MsrAB is required for bacterial virulence and colonization. (A) Presented is an experimental scheme of a mouse model of preterm birth. CF-1 mice were bred at a ratio of 2:1 (female-male) (day 0). On day 16 or 17 of gestation, groups of 6 female mice were infected with F. nucleatum (5 × 10^7^ CFU) via tail vein injection. The number of live and dead pups was recorded during the next 7 days. (B) The parental, *msrAB*, and *ccdA-trx* mutant strains were used in the mouse model of preterm birth as described for panel A. Pup survival (percent) was determined accordingly. Significance was analyzed by the Mantel-Cox log-rank test with GraphPad; ***, *P* < 0.001. (C and D) Groups of 3 pregnant mice (see panel A) were used in the colonization experiment. At 6, 24, 48, and 24 h postinfection, liver, spleen, placenta, amniotic fluid (AF), and fetus from individual mice were harvested and homogenized for bacterial enumeration (CFU). Significance was analyzed by two-way analysis of variance; ***, *P* < 0.001.

We then evaluated the role of MsrAB in bacterial virulence using a mouse model of preterm birth as developed by Han and colleagues (17) (Fig. 5A). All mice challenged with the parental fusobacterial strain gave birth to stillborn pups within 72 h (Fig. 5B, black circles). In sharp contrast, pups survived when the mother mice were infected with the Δ*msrAB* strain, with normal-sized litters born between day 20 and 23 (Fig. 5B, red squares). We also tested a mutant devoid of both *trx* and *ccdA* (Δ*trx* Δ*ccdA*) in this preterm birth model. As predicted, the double mutant strain exhibited the same attenuated phenotype as the Δ*msrAB* mutant (Fig. 5B, blue triangles). Compared with the parent strain, the Δ*msrAB* and the Δ*trx* Δ*ccdA* mutant strains showed no significant defect in bacterial cell growth in rich media (Fig. S3). Clearly, MsrAB and the associated factors Trx/CcdA are major determinants of fusobacterial virulence, as evidenced in the murine model of preterm birth.

10.1128/mbio.03022-21.3 FIG S3 Determination of bacterial growth and number of CFU in F. nucleatum strains. (A and B) Overnight cultures of indicated strains were used to inoculate fresh cultures with starting OD_600_ of 0.1 in an anaerobic chamber. Bacterial growth was monitored by OD_600_ every 2 h. Arrowheads indicate three time points (8, 16, and 24 h), at which aliquots were taken for bacterial numeration (CFU) as presented in panel B. All experiments were performed in triplicate in three biological repeats. Statistical analysis was performed with GraphPad (n.s., not significant). Download FIG S3, PDF file, 0.1 MB. Copyright © 2022 Scheible et al. 2022 Scheible et al. https://creativecommons.org/licenses/by/4.0/ This content is distributed under the terms of the Creative Commons Attribution 4.0 International license.

To test whether the failure of the tail vein-injected Δ*msrAB* mutant to induce preterm birth shown above was due to its inability to disseminate to and colonize the placenta, we sacrificed infected mice at 6, 24, 48, and 72 h postinfection and harvested liver, spleen, placenta, amniotic fluid (AF), and fetal tissue. The tissue of each organ was homogenized, and the bacterial load was enumerated. Within 24 h, fusobacterial cells were detected in the liver and spleen of mother mice infected with either the parental strain or the Δ*msrAB* mutant (Fig. 5C and D, liver and spleen). Within 72 h, the liver and spleen cleared the bacteria. Unlike the Δ*msrAB* mutant, the parental strain spread to the fetal tissue. The parent strain displayed a robust homing affinity for the placenta and amnion, appearing in detectable quantities within 24 h and expanding and spreading to fetus over the remainder of the experiment (Fig. 5C, placenta, AF, and fetus). Strikingly, the Δ*msrAB* mutant was scarcely detected in the placenta and AF, appearing late (48 h), and was hardly detected in fetus during the entire length of the experiment (Fig. 5D). We conclude that the methionine sulfoxide reductase MsrAB is critical for fusobacterial colonization and pathogenicity in the placenta.

## DISCUSSION

Rapid and controlled reprogramming of gene expression is central to the ability of bacteria to adapt to and thrive in changing and hostile environments in natural habitats or within animal hosts. In this paper, we describe a key genetic mechanism by which an obligate anaerobe, F. nucleatum, survives oxidative stress and the importance of this mechanism in success as a pathogen. F. nucleatum is a well-established human commensal organism frequently found as an abundant member of the oral microbiome that has been associated with several distinct human pathologies involving both oral and extra-oral sites, including the respiratory tract, colon, and placenta (4). To date, only a small number of factors contributing to its pathogenicity have been identified, and very little is known about their gene regulation. Lack of knowledge about gene regulation may reflect the inherent difficulty of culturing fastidious anaerobes and the hitherto underdeveloped genetic tractability of fusobacteria. Recent advancements in bacterial genomics and genetic manipulations of this organism, however, have begun to facilitate fruitful molecular studies (36–38). We were then able to undertake a genetic investigation of adaptive response in fusobacteria. We discovered that a fused methionine sulfoxide reductase, MsrAB, is a key factor for oxidative stress defense and critical for fusobacterial colonization and virulence. Furthermore, we report the important finding that the stress-induced enzyme MsrAB and two accessory components (Trx and CcdA) are regulated by a genetically linked two-component signal transduction system (ModRS), which acts as a global regulator of a vast number of genes that constitute a complex oxidative stress defense system for this “commensal-turned-pathogen.”

We adapted two complementary approaches, probing candidate genes and carrying out a global transcriptome analysis, to investigate the oxidative stress defense system in fusobacteria. Although genes coding for methionine sulfoxide reductases are often clustered with *trx* and *ccdA* genes, which also encode important antioxidant pathway proteins, these genetic elements are infrequently reported to be heritably linked to a TCS in the same locus (31, 32). The close proximity of these genes, *ccdA*, *trx*, *msrAB*, *modR*, and *modS*, suggests that they are functionally interconnected in F. nucleatum. Indeed, we show here that all these genes in the *msrAB* locus are upregulated under oxidative stress (Fig. 1). We demonstrated further that ModRS modulate expression of not only linked *msrAB*, *trx*, and *ccdA* genes (Fig. 1) but also a large number of metabolism and transport genes outside the *msrAB* locus (Tables 1 and 2 and Fig. 2). The response regulator ModR appears to be an activator of *trx* and *ccdA*, since deletion of *modR* decreases expression of those two genes (Fig. 1F). In contrast, in the absence of the sensor protein ModS, ModR appears to be a repressor of *trx* and *ccdA* (Fig. 1F). ModR contains a conserved aspartate residue, D55, predicted to be subjected to phosphorylation by ModS. The phosphorylation status of ModR is important to gene regulation. A mutation that abrogates phosphorylation (D55A) caused a significant reduction of the expression of *trx* and *ccdA*, whereas a control mutation that mimics phosphorylation (D55E) was without effect (see Fig. S1 in the supplemental material). Future experiments need to address how ModS influences expression of *msrAB*, *trx*, and *ccdA*.

Our genome-wide transcriptome analysis utilizing the *modR* deletion mutant revealed that ModR is a global regulator of gene expression that acts as both an activator and a repressor of many specific genes that constitute a large regulon in fusobacteria. Other regulatory targets of the response regulator ModR include the most upregulated genes encoding an ethanolamine utilization (Eut) system (Table S1). Genes coding for proteins related to a phosphotransferase system (PTS) are the most downregulated (Table S1). Found in many bacterial species, including *Enterococcus*, Escherichia, *Clostridium*, *Listeria*, and Salmonella, the Eut systems enable bacteria to utilize and catabolize environmental ethanolamine, a breakdown product of the membrane lipid phosphatidylethanolamine that serves as a carbon and nitrogen source (39). On the other hand, the PTS enables sugar response, transport, and phosphorylation (40). While virtually nothing is known about ethanolamine utilization in F. nucleatum, a gene locus coding for an Eut system has been identified (41). Intriguingly, given that the F. nucleatum Eut gene locus encodes a TCS (41) and ModRS modulates expression of many Eut genes, cross-regulation by these two-component transduction systems is likely in F. nucleatum. Curiously, first trimester placental villi contain abundant phosphoethanolamine, and third trimester villi contain large amounts of ethanolamine (42), suggesting the intriguing possibility that ModR/ModS function to respond to metabolic signals present in the placental environment. In the placenta, fusobacteria must simultaneously cope with oxidative attacks from a plethora of immune and epithelial cells.

As a human commensal and a pathogen, fusobacteria must face antibacterial reactive oxygen species that are abundantly produced from intestinal epithelial and immune cells (43, 44). Thus, a potential role of fusobacterial MsrAB and the associated factors might be a physiological defense against the oxidative stress. Indeed, our work demonstrated that mutants lacking *modR*, *msrAB*, *trx*, or *ccdA* were all sensitive to hydrogen peroxide (Fig. 3A and B). Since *msrAB*, *trx*, and *ccdA* were upregulated in the *modS* mutant (Fig. 1), it is not surprising to observe that the *modS* mutant displayed the same level of oxidative stress tolerance as the parental strain (Fig. 3B). However, survival of fusobacteria in macrophages was severely affected in the absence of MsrAB (Fig. 3C), and mutants devoid of *modS*, *modR*, *msrAB*, *trx*, and *ccdA* were all defective in adherence to and invasion of colorectal cancer cells, regardless of cell lines (Fig. 4). The adherence/invasion defects of the Δ*trx* and Δ*ccdA* mutants with the HT-29 cells, however, were not as robust as that of the HCT116 cells, although the nature of this cell line-to-cell line variation is presently unknown.

How are MsrAB and its associated factors involved in adherence/invasion and virulence? Since methionine sulfoxide reductases function to repair oxidized methionine residues in various proteins by reducing them to methionine (34), the absence of the methionine sulfoxide reductase MsrAB likely contributes to inadequate repair of proteins such as Fap2, FadA, and RadD (19–22, 45) under oxidative stress, reducing fusobacterial adhesion/invasion and virulence. It is noteworthy that *fadA* and *fap2* mutants are defective in placental colonization (19, 22). It is challenging, however, to determine which proteins are direct targets of MsrAB, as all proteins contain at least one methionine residue, and it is unclear which positions of methionine are prone to oxidation in the target proteins. Since Trx and CcdA are known to maintain the reductase activity of MsrAB (32, 46), the defective phenotypes of the *trx* and *ccdA* mutants may be directly associated with the MsrAB activity. In support of this conjecture, the double mutant lacking both *trx* and *ccdA* is attenuated in virulence in the mouse preterm birth model, as observed with the *msrAB* mutant (Fig. 5B), which exhibits significantly reduced colonization of the placenta, amniotic fluid, and fetus (Fig. 5C and D).

Taken together, our work presented here reveals for the first time that the methionine sulfoxide reductase MsrAB plays a critical role in fusobacterial oxidative stress defense, adherence/invasion, and virulence. In addition, the molecular linkage of MsrAB, its activators Trx and CcdA, and the TCS ModRS may contribute to the shift of F. nucleatum from oral commensal to distal pathogen. The full details of ModR-mediated regulation remain to be investigated and may have implications in all bacteria.

## MATERIALS AND METHODS

### Bacterial strains, plasmids, media, and growth.

Bacterial strains and plasmids used in this study are listed in Table S5 in the supplemental material. F. nucleatum was grown in tryptic soy broth supplemented with 1% Bacto peptone plus 0.25% freshly made cysteine (TSPC) or on TSPC agar plates with 1% vitamin K1-hemin solution or BBL Columbia agar with 5% sheep blood in an anaerobic chamber (5% CO_2_, 2.5% H_2_, and 92.5% N_2_), as previously described (38). When necessary, chloramphenicol (15 μg/mL) or thiamphenicol (5 μg/mL) was added. Reagents were purchased through Sigma-Aldrich.

10.1128/mbio.03022-21.6 TABLE S3 Exclusively expressed genes in the Δ*modR* mutant exposed to hydrogen peroxide compared to the treated parental strain relative to the untreated parental strain. Download Table S3, DOCX file, 0.01 MB. Copyright © 2022 Scheible et al. 2022 Scheible et al. https://creativecommons.org/licenses/by/4.0/ This content is distributed under the terms of the Creative Commons Attribution 4.0 International license.

10.1128/mbio.03022-21.7 TABLE S4 Exclusively expressed genes in the parental strain exposed to hydrogen peroxide compared to the Δ*modR* mutant, relative to the untreated parental strain. Download Table S4, DOCX file, 0.02 MB. Copyright © 2022 Scheible et al. 2022 Scheible et al. https://creativecommons.org/licenses/by/4.0/ This content is distributed under the terms of the Creative Commons Attribution 4.0 International license.

10.1128/mbio.03022-21.8 TABLE S5 Strains and plasmids used in this study. Download Table S5, DOCX file, 0.02 MB. Copyright © 2022 Scheible et al. 2022 Scheible et al. https://creativecommons.org/licenses/by/4.0/ This content is distributed under the terms of the Creative Commons Attribution 4.0 International license.

To monitor bacterial growth, overnight cultures of individual F. nucleatum strains were used to inoculate fresh 10-mL cultures in TSPC with a starting OD_600_ of ∼0.1. Bacterial growth in an anaerobic chamber at 37°C was monitored by taking the OD_600_ every 2 h for 24 h. In parallel, 0.1-mL aliquots of these cultures were collected every 8 h for bacterial numeration (CFU) on TSPC agar plates.

### Genetic manipulations in F. nucleatum.

Deletion mutants of F. nucleatum and their complementing strains (Table S5) were generated according to published protocols (37, 38), with primers used for these constructions listed in Table S6.

10.1128/mbio.03022-21.9 TABLE S6 Primers used in this study. Download Table S6, DOCX file, 0.02 MB. Copyright © 2022 Scheible et al. 2022 Scheible et al. https://creativecommons.org/licenses/by/4.0/ This content is distributed under the terms of the Creative Commons Attribution 4.0 International license.

To generate plasmids expressing ModR_D55E_ or ModR_D55A_ by site-directed mutagenesis, a DNA region encompassing the *modR* gene and its promoter from pModR (Table S5) were subcloned into pCGL0243 (47) before site-directed mutagenesis by inverse PCR was performed as previously described (48). With pCGL0243-ModR as the template and a pair of phosphorylated forward and reverse primers carrying desired mutations (Table S6), linear mutant plasmids were PCR amplified. The amplified plasmids were circularized by T4 ligase before introducing them to E. coli DH5α. DNA sequencing was used to confirm the point mutations in the cloned fragments, which were then subcloned into pCWU6 (Table S6) prior to introducing them to fusobacteria.

### Real-time PCR.

The real-time PCR experiment was performed as previously described (48). Briefly, total RNA was extracted from F. nucleatum cells grown in TSPC overnight. Cultures were normalized to an OD_600_ of 1.0. Cell pellets were washed once with PBS and then resuspended in RLT buffer (Qiagen) and lysed using a bead beater. The RNeasy minikit (Qiagen) was used to extract nucleic acids from the samples. All samples were treated with DNase (Qiagen), and then the RNeasy MinElute cleanup kit (Qiagen) was used to purify RNA; 500 ng of purified RNA was used to make cDNA using random primers (Invitrogen) and Moloney murine leukemia virus (MMLV) reverse transcriptase (Invitrogen) as described by the manufacturer's protocol; a no MMLV control was prepared with 500 ng of purified RNA simultaneously to serve as a control for checking DNA contamination. Primers, RT-16S-F and RT-16S-R (Table S6), were used in a PCR using Apex 2× Taqmix (Genesse Scientific) with either cDNA or a no MMLV control to guarantee the pure quality of extracted RNA for this quantitative PCR. cDNA samples were used for quantitative PCR, with appropriate primers listed in Table S6, by using iTAQ SYBR green supermix (Bio-Rad) and the CFX96 Touch real-time PCR detection system (Bio-Rad). The data are presented as relative values normalized to a reference gene (16S RNA) and untreated samples.

### Gene expression profiling by RNA-seq.

RNA samples from the parental and Δ*modR* mutant strains treated with or without 1 mM H_2_O_2_ were prepared according to a published protocol (48). Briefly, using RNeasy Mini kits (Qiagen) according to the manufacturer’s protocol, bacterial cell pellets were harvested from 3 mL log-phase culture and resuspended into RNA-free TE buffer (10 mM Tris-HCl, pH 8, 1 mM EDTA). Cells were lysed, and supernatants obtained by centrifugation were used for RNA purification. Purified RNA was treated with DNase (Qiagen) and cleaned by using an RNeasy cleanup kit (Qiagen). DNA-free quality of extracted RNA was checked using a no MMLV control as described above for the real-time PCR method. Quality of RNA samples was determined using an Agilent 2100 Bioanalyzer (Agilent Technologies). Samples with RNA integrity number (RIN) values greater than 8 were used for RNA-seq.

cDNA libraries were prepared for RNA-seq, and sequencing was carried out in the paired-end mode on an Illumina HiSeq as previously described (49). Using DESEq2, processing and mapping of the paired-end reads and differential gene expression analysis were performed as previously reported (49). A cutoff of log_2_ (fold change) of ±1.0 was considered for genes differentially transcribed under the examined conditions.

### Reactive oxygen sensitivity.

To explore the sensitivity of F. nucleatum to reactive oxygen species, log-phase cultures of fusobacteria in TSPC were diluted to an OD_600_ of 0.1. H_2_O_2_ was added to final concentrations of 0.5, 1, or 1.5 mM. The same procedure was followed with NaOCl, instead adding to final concentrations of 5, 10, or 20 mM. Bacteria were allowed to grow for 24 h, and growth was measured by OD_600_. Three independent experiments were performed, with each in triplicates. Significance was determined by applying Student's *t* test (***, *P* < 0.001; **, *P* < 0.01).

### Macrophage survival assay.

The ability of F. nucleatum strains to survive in macrophage was determined by culturing RAW 264.7 cells in Dulbecco’s modification of Eagle’s medium (DMEM) supplemented with 10% FBS and 1% penicillin-streptomycin. Macrophage cells were cultured to 80% confluence and challenged with individual F. nucleatum strains, grown to mid-log phase in TSPC, for 0, 30, or 60 min at a multiplicity of infection (MOI) of 50. Cells were washed with water and plated on TSPC plates for bacterial numeration. CFU numbers were recorded and normalized against the parental strain treated with immediate exposure to macrophage media. The results were presented as fold change from 3 experimental and technical replicates, and significance was determined by Student's *t* test (***, *P* < 0.001; **, *P* < 0.01; *, *P* < 0.05).

### Adherence and invasion of colorectal cancer cells.

HCT116 and HT-29 colorectal cancer cell lines (American Type Culture Collection [ATCC]) were grown in Dulbecco’s modification of Eagle’s medium (DMEM) supplemented with 10% FBS and 1% penicillin-streptomycin. Mammalian cells were cultured to 80% confluence and challenged with individual F. nucleatum strains grown to mid-log phase in TSPC at an MOI of 100. Bacterial cells were allowed to attach or invade the mammalian cells for 90 or 180 min before being gently washed to remove unattached cells. For invasion, parallel samples were treated with gentamicin and metronidazole (300 and 200 μg/mL, respectively) to kill extracellular bacteria. Bacteria from all groups were then collected by lysing mammalian cells with cold distilled water. Bacterial cells were serially diluted on TSPC plates and CFU numbers were recorded. The results are presented as averages from at least 3 experimental and technical replicates with the level of the parental strain set to 1, with significance determined by Student's *t* test (***, *P* < 0.001; **, *P* < 0.01; *, *P* < 0.05).

### Bacterial infection *in vivo*.

To evaluate fusobacterial virulence *in vivo*, a mouse model of preterm birth was employed (17, 38). Briefly, 10-week-old CF-1 mice, purchased from Charles Rivers Laboratories, were mated at the female-to-male ratio of 2:1. On day 16 or 17 of gestation, pregnant mice were infected via tail vein injection with ∼5 × 10^7^ CFU of individual fusobacterial strains suspended in Dulbecco's phosphate-buffered saline (DPBS). The number of live and stillborn pups was recorded for the next 7 days. Statistical analyses were carried out relative to the parental strain, and significance was determined via Mantel-Cox testing (***, *P* < 0.001).

To examine bacterial colonization in different organs, the same procedure as that described above was carried out, except that animals were sacrificed at 6, 24, 48, or 72 h postinfection. Liver, spleen, placenta, amniotic fluid, and fetus were collected, homogenized, and serially diluted onto TSPC plates for bacterial enumeration. After 48 h of growth, CFU were counted and recorded. All animal procedures were approved by the UCLA Animal Research Committee.

### Data availability.

The RNA-seq data were deposited in the NCBI Gene Expression Omnibus (GEO) database with the accession number of GSE174320.
