# Supplementary figures and images for: The Fused Methionine Sulfoxide Reductase MsrAB Promotes Oxidative Stress Defense and Bacterial Virulence in Fusobacterium nucleatum
Source: mBio. 2022 Apr 14;13(3):e03022-21. doi: 10.1128/mbio.03022-21 (PMC9239216; doi:10.1128/mbio.03022-21)

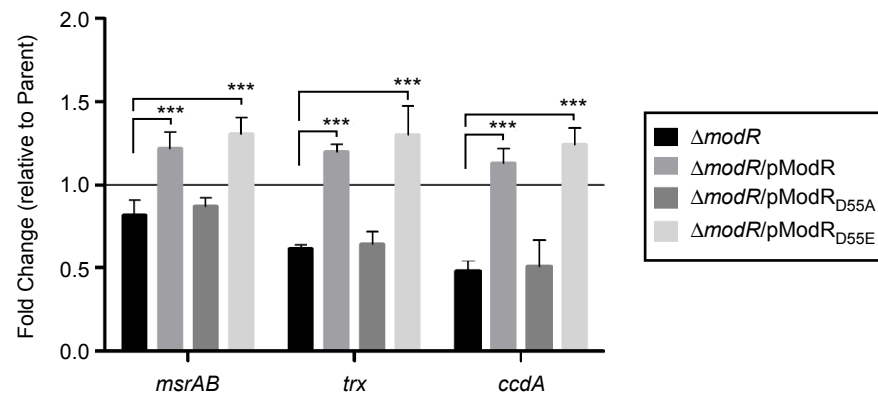

Figure S1: Scheible et al.

Supplement: FIG S1 [file mbio.03022-21-s0003.pdf]

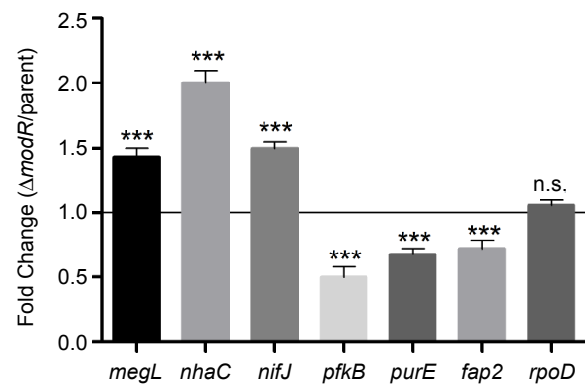

Figure S2: Scheible et al.

Supplement: FIG S2 [file mbio.03022-21-s0004.pdf]

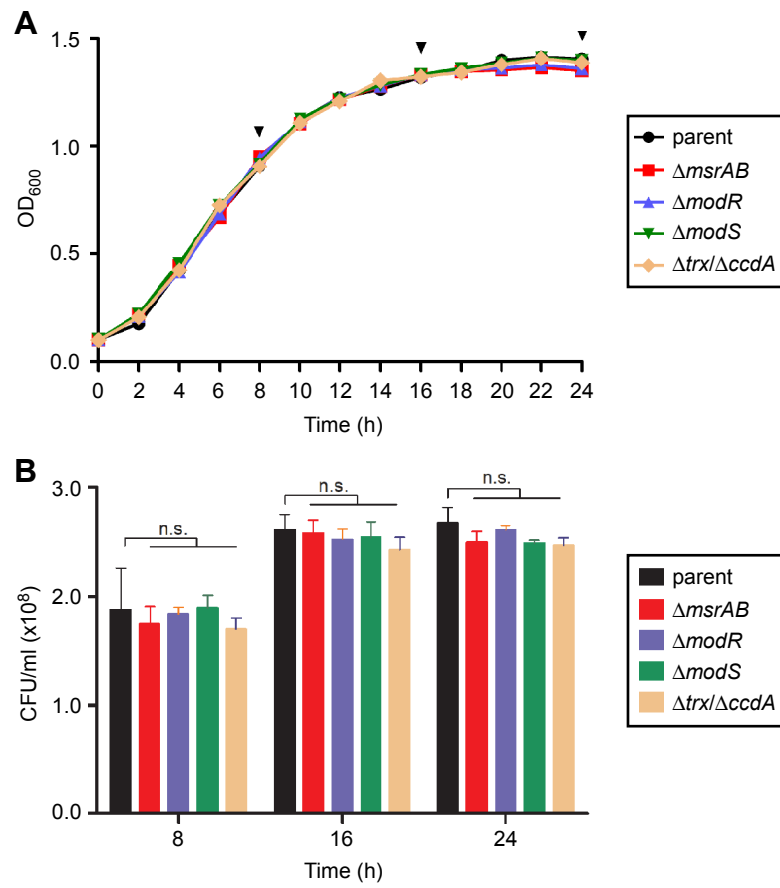

Figure S3: Scheible et al.

Supplement: FIG S3 [file mbio.03022-21-s0005.pdf]
