# Supplementary material for: The Fused Methionine Sulfoxide Reductase MsrAB Promotes Oxidative Stress Defense and Bacterial Virulence in Fusobacterium nucleatum
Source: mBio. 2022 Apr 14;13(3):e03022-21. doi: 10.1128/mbio.03022-21 (PMC9239216; doi:10.1128/mbio.03022-21)
Supplement: TABLE S3 [file mbio.03022-21-s0006.docx]

**Table S3:** Exclusively expressed genes in the Δ*modR* mutant exposed to hydrogen peroxide as compared to the treated parental strain, relative to the untreated parental strain

| **Locus ID** | **Gene** | **Predicted Function** | **Fold change** |
| --- | --- | --- | --- |
|  |  | ***Upregulated genes*** |  |
| HMPREF0397_RS02050 | *grpE* | nucleotide exchange factor GrpE | 2.94 |
| HMPREF0397_RS02055 | *dnaK* | molecular chaperone DnaK | 2.61 |
| HMPREF0397_RS06435 |  | DUF1667 domain-containing protein | 2.52 |
| HMPREF0397_RS09720 |  | hypothetical protein | 2.45 |
| HMPREF0397_RS05695 |  | hypothetical protein | 2.42 |
| HMPREF0397_RS02060 |  | methylated-DNA--[protein]-cysteine S-methyltransferase | 2.32 |
| HMPREF0397_RS06440 |  | FAD-dependent oxidoreductase | 2.24 |
| HMPREF0397_RS10165 |  | WYL domain-containing protein | 2.10 |
| HMPREF0397_RS08325 |  | toxin-antitoxin system YwqK family antitoxin | 2.08 |
| HMPREF0397_RS05710 |  | hypothetical protein | 2.05 |
| HMPREF0397_RS01025 | *rpsR* | 30S ribosomal protein S18 | 2.04 |
| HMPREF0397_RS03170 | *rnmV* | ribonuclease M5 | 2.03 |
| HMPREF0397_RS05610 |  | DNA starvation/stationary phase protection protein | 2.03 |
|  |  | ***Downregulated Genes*** |  |
| HMPREF0397_RS08130 |  | C4-dicarboxylate ABC transporter | -2.07 |
| HMPREF0397_RS0110390 |  | redoxin family protein | -2.07 |
| HMPREF0397_RS00415 |  | DUF4261 domain-containing protein | -2.09 |
| HMPREF0397_RS04915 |  | hypothetical protein | -2.11 |
