# Supplementary material for: The Fused Methionine Sulfoxide Reductase MsrAB Promotes Oxidative Stress Defense and Bacterial Virulence in Fusobacterium nucleatum
Source: mBio. 2022 Apr 14;13(3):e03022-21. doi: 10.1128/mbio.03022-21 (PMC9239216; doi:10.1128/mbio.03022-21)
Supplement: TABLE S4 [file mbio.03022-21-s0007.docx]

**Table S4:** Exclusively expressed genes in the parental strain exposed to hydrogen peroxide as compared to the Δ*modR* mutant, relative to the untreated parental strain

| **Locus ID** | **Gene** | **Predicted Function** | **Fold change** |
| --- | --- | --- | --- |
| ***Upregulated genes*** |  |  |  |
| HMPREF0397_RS07360 |  | imidazolonepropionase | 4.95 |
| HMPREF0397_RS07355 |  | formiminotetrahydrofolate cyclodeaminase | 4.86 |
| HMPREF0397_RS03630 | *hutH* | histidine ammonia-lyase | 4.52 |
| HMPREF0397_RS04970 |  | formate--tetrahydrofolate ligase | 3.87 |
| HMPREF0397_RS09270 |  | GntP family permease | 3.26 |
| HMPREF0397_RS09275 | *dsdA* | D-serine ammonia-lyase | 2.91 |
| HMPREF0397_RS09280 |  | amino-acid racemase | 2.87 |
| HMPREF0397_RS01790 |  | helix-turn-helix transcriptional regulator | 2.74 |
| HMPREF0397_RS01795 |  | CoA-disulfide reductase | 2.74 |
| HMPREF0397_RS07550 |  | CAP domain-containing protein | 2.63 |
| HMPREF0397_RS08715 |  | metal ABC transporter permease | 2.60 |
| HMPREF0397_RS00980 |  | hypothetical protein | 2.57 |
| HMPREF0397_RS10895 |  | hypothetical protein | 2.52 |
| HMPREF0397_RS02250 |  | RloB domain-containing protein | 2.44 |
| HMPREF0397_RS04360 |  | hypothetical protein | 2.43 |
| HMPREF0397_RS06500 |  | ABC transporter ATP-binding protein | 2.42 |
| HMPREF0397_RS01990 |  | ferredoxin--NADP reductase | 2.37 |
| HMPREF0397_RS04375 |  | hypothetical protein | 2.34 |
| HMPREF0397_RS09555 |  | ABC transporter ATP-binding protein | 2.31 |
| HMPREF0397_RS01310 |  | hypothetical protein | 2.31 |
| HMPREF0397_RS02255 |  | adenylate cyclase | 2.31 |
| HMPREF0397_RS06340 |  | MATE family efflux transporter | 2.30 |
| HMPREF0397_RS04795 |  | AAA family ATPase | 2.29 |
| HMPREF0397_RS06495 |  | ABC transporter permease | 2.26 |
| HMPREF0397_RS08955 |  | N-glycosylase/DNA lyase | 2.26 |
| HMPREF0397_RS07285 |  | tRNA threonylcarbamoyladenosine dehydratase | 2.25 |
| HMPREF0397_RS04170 |  | ABC transporter ATP-binding protein | 2.24 |
| HMPREF0397_RS04165 |  | MATE family efflux transporter | 2.23 |
| HMPREF0397_RS09565 |  | ABC transporter substrate-binding protein | 2.21 |
| HMPREF0397_RS07390 |  | helix-turn-helix domain-containing protein | 2.21 |
| HMPREF0397_RS05530 |  | amidohydrolase | 2.19 |
| HMPREF0397_RS08720 |  | metal ABC transporter ATP-binding protein | 2.18 |
| HMPREF0397_RS01985 |  | inorganic pyrophosphatase | 2.17 |
| HMPREF0397_RS01875 |  | TolC family protein | 2.17 |
| HMPREF0397_RS05000 |  | fimbrial assembly protein | 2.17 |
| HMPREF0397_RS08085 |  | IclR family transcriptional regulator | 2.16 |
| HMPREF0397_RS06345 |  | 5-methyltetrahydrofolate--homocysteine methyltransferase | 2.15 |
| HMPREF0397_RS03660 |  | fructose-bisphosphatase class III | 2.15 |
| HMPREF0397_RS01035 |  | DUF1858 domain-containing protein | 2.15 |
| HMPREF0397_RS08730 |  | MATE family efflux transporter | 2.14 |
| HMPREF0397_RS08725 |  | zinc ABC transporter solute-binding protein | 2.14 |
| HMPREF0397_RS02390 |  | ABC transporter permease | 2.14 |
| HMPREF0397_RS02395 |  | ABC transporter permease | 2.12 |
| HMPREF0397_RS02835 |  | iron-containing alcohol dehydrogenase | 2.10 |
| HMPREF0397_RS01045 |  | MATE family efflux transporter | 2.09 |
| HMPREF0397_RS04790 |  | DNA polymerase IV | 2.08 |
| HMPREF0397_RS04820 |  | hypothetical protein | 2.08 |
| HMPREF0397_RS05785 |  | DUF1007 family protein | 2.07 |
| HMPREF0397_RS02770 |  | ABC transporter ATP-binding protein | 2.05 |
| HMPREF0397_RS03280 |  | hypothetical protein | 2.05 |
| HMPREF0397_RS03650 |  | helix-turn-helix transcriptional regulator | 2.05 |
| HMPREF0397_RS03830 |  | ATP-binding protein | 2.04 |
| HMPREF0397_RS01305 |  | DUF2247 family protein | 2.04 |
| HMPREF0397_RS09560 |  | ABC transporter permease | 2.02 |
| HMPREF0397_RS05875 |  | hypothetical protein | 2.02 |
| HMPREF0397_RS03340 | *cadA* | cadmium-translocating P-type ATPase | 2.01 |
| HMPREF0397_RS07065 |  | DUF1858 domain-containing protein | 2.01 |
| HMPREF0397_RS01555 |  | hypothetical protein | 2.01 |
| ***Downregulated Genes*** |  |  |  |
| HMPREF0397_RS02290 |  | ubiquinone/menaquinone biosynthesis methyltransferase | -2.00 |
| HMPREF0397_RS09900 |  | diguanylate phosphodiesterase | -2.00 |
| HMPREF0397_RS05960 |  | dicarboxylate/amino acid:cation symporter | -2.01 |
| HMPREF0397_RS08310 | *citE* | citrate (pro-3S)-lyase subunit beta | -2.02 |
| HMPREF0397_RS05415 |  | hypothetical protein | -2.02 |
| HMPREF0397_RS02355 |  | adhesion protein FadA | -2.03 |
| HMPREF0397_RS02335 |  | electron transfer flavoprotein subunit beta/FixAfamily protein | -2.03 |
| HMPREF0397_RS00575 |  | sodium:alanine symporter family protein | -2.05 |
| HMPREF0397_RS02285 |  | FAD-dependent oxidoreductase | -2.06 |
| HMPREF0397_RS09895 |  | M42 family metallopeptidase | -2.06 |
| HMPREF0397_RS02350 |  | LrgB family protein | -2.07 |
| HMPREF0397_RS05405 |  | hypothetical protein | -2.12 |
| HMPREF0397_RS02330 |  | acyl-CoA dehydrogenase | -2.13 |
| HMPREF0397_RS02480 | *ggt* | gamma-glutamyltransferase | -2.14 |
| HMPREF0397_RS07830 | *fap2* | galactose-inhibitable autotransporter adhesin Fap2 | -2.14 |
| HMPREF0397_RS08295 |  | oxaloacetate decarboxylase subunit alpha | -2.16 |
| HMPREF0397_RS02485 |  | hypothetical protein | -2.17 |
| HMPREF0397_RS02365 |  | hypothetical protein | -2.20 |
| HMPREF0397_RS00145 |  | hypothetical protein | -2.26 |
| HMPREF0397_RS06255 |  | hypothetical protein | -2.27 |
| HMPREF0397_RS04050 |  | ABC transporter permease | -2.37 |
| HMPREF0397_RS02340 |  | electron transfer flavoprotein subunit alpha/FixB family protein | -2.38 |
| HMPREF0397_RS04055 |  | hypothetical protein | -2.39 |
| HMPREF0397_RS02370 | *radD* | autotransporter adhesin RadD | -2.45 |
| HMPREF0397_RS04045 |  | ABC transporter | -2.59 |
