# Supplementary material for: The Fused Methionine Sulfoxide Reductase MsrAB Promotes Oxidative Stress Defense and Bacterial Virulence in Fusobacterium nucleatum
Source: mBio. 2022 Apr 14;13(3):e03022-21. doi: 10.1128/mbio.03022-21 (PMC9239216; doi:10.1128/mbio.03022-21)
Supplement: TABLE S5 [file mbio.03022-21-s0008.docx]

**Table S5:** Strains and plasmids used in this study

| **Strain and Plasmid** | **Description** | **Reference** |
| --- | --- | --- |
| ***Strain*** |  |  |
| *F. nucleatum* ATCC 23726 | Urogentical tract isolate | (1) |
| *F. nucleatum* CW1 | Isogenic derivative of 23726; lacking *galK* | (1) |
| ∆*modS* | Isogenic derivative of CW1, lacking *modS* | This study |
| *∆modR* | Isogenic derivative of CW1, lacking *modR* | This study |
| *∆msrAB* | Isogenic derivative of CW1, lacking *msrAB* | This study |
| *∆trx* | Isogenic derivative of CW1, lacking *trx* | This study |
| *∆ccdA* | Isogenic derivative of CW1, lacking *ccdA* | This study |
| *∆ccdA*/*∆trx* | Isogenic derivative of CW1, lacking *ccdA* and *trx* | This study |
| ***Plasmid*** |  |  |
| pCWU6 | Derivative of pHS30 | (1) |
| pCM-galK | *Clostridium perfringens* vector expressing *galK* | (1) |
| pCGL243 | Cloning vector | (2) |
| p∆*modS* | Derivative of pCM-galK; deletion vector of *modS* | This study |
| p∆*modR* | Derivative of pCM-galK; deletion vector of *modR* | This study |
| p∆*trx* | Derivative of pCM-galK; deletion vector of *trx* | This study |
| p∆*ccdA* | Derivative of pCM-galK; deletion vector of *ccdA* | This study |
| pModS | pCWU6 expressing ModS | This study |
| pModR | pCWU6 expressing ModR | This study |
| pModR_D55E_ | pCWU6 expressing ModR with D55E mutation | This study |
| pModR_D55A_ | pCWU6 expressing ModR with D55A mutation | This study |
| pMsrAB | pCWU6 expressing MsrAB | This study |
| pTrx | pCWU6 expressing Trx | This study |
| pCcdA | pCWU6 expressing CcdA | This study |

References

1. Wu C, Al Mamun AAM, Luong TT, Hu B, Gu J, Lee JH, D'Amore M, Das A, Ton-That H. 2018. Forward Genetic Dissection of Biofilm Development by Fusobacterium nucleatum: Novel Functions of Cell Division Proteins FtsX and EnvC. mBio 9.

2. Ton-That H, Schneewind O. 2003. Assembly of pili on the surface of Corynebacterium diphtheriae. Mol Microbiol 50:1429-38.
