# Supplementary material for: The Fused Methionine Sulfoxide Reductase MsrAB Promotes Oxidative Stress Defense and Bacterial Virulence in Fusobacterium nucleatum
Source: mBio. 2022 Apr 14;13(3):e03022-21. doi: 10.1128/mbio.03022-21 (PMC9239216; doi:10.1128/mbio.03022-21)
Supplement: TABLE S6 [file mbio.03022-21-s0009.docx]

**Table S6:** Primers used in this study

| **Primer** | **Sequence ^a^** | **Application** |
| --- | --- | --- |
| modS-F-A-BamHI | GGCGGGATCCGAGGGGTATAAAACAACTAATAG | ∆*modS* |
| modS-R-A-HindIII | GGCGAAGCTTCTTGAACTGAAATAAAATATACTTCC | ∆*modS* |
| modS-F-B-HindIII | GGCGAAGCTTGTTATTTCTATTACAAAGGCTTTATC | ∆*modS* |
| modS-R-B-SalI | GGCGGTCGACGAATCAAATATATCTTTGGTTAAAGCC | ∆*modS* |
| modR-F-A-SacI | AGAGGAGCTCCTATGGTGTAATAGATGTAACTTC | ∆*modR* |
| modR-R-A-KpnI | AGAGGGTACCCTATTAGTTGTTTTATACCCCTC | ∆*modR* |
| modR-F-B-KpnI | AGAGGGTACCGTTCAGGGTATTTAAGTATTATG | ∆*modR* |
| modR-R-B-SalI | AGAGGTCGACCAAACAACTTATAACTATCATTTC | ∆*modR* |
| msrAB-F-A-BamHI | GGCGGGATCCGGTGGTTTGTTGATAATTATTATGGG | ∆*msrAB* |
| msrAB-R-A-KpnI | GGCGGGTACCCTTGCATCATACTTAACATGGAC | ∆*msrAB* |
| msrAB-F-B-KpnI | GGCGGGTACCGATGATGGGCCAAGATAGAG | ∆*msrAB* |
| msrAB-R-B-SalI | GGCGGTCGACCTGTCTTAGCTGAAATTTCTTTCTG | ∆*msrAB* |
| Trx-F-A-SacI | GGCGGAGCTCGGAGTATAAGAAATGTTAAATACAGAG | ∆*trx* |
| Trx-R-A-KpnI | GGCGGGTACCGACAAATTGGACACCAAGAAGC | ∆*trx* |
| Trx-F-B-KpnI | GGCGGGTACCGAATGATGCTCATATGATGAAAGATG | ∆*trx* |
| Trx-R-B-SalI | GGCGGTCGACCTCTATCTCTTGGCCCATCATC | ∆*trx* |
| ccdA-F-A-SacI | GGCGGAGCTCCTAGATAATCTAAATAGCTTGCTTTC | ∆*ccdA* |
| ccdA-R-A-KpnI | GGCGGGTACCCTCTGTATTTAACATTTCTTATACTCC | ∆*ccdA* |
| ccdA-F-B-KpnI | GGCGGGTACCGTTTAGCGACACCTTTTGTCATTC | ∆*ccdA* |
| ccdA-R-B-SalI | GGCGGTCGACCATCTTTCAATCATATGAGCATCATTC | ∆*ccdA* |
| ModR-D55E-F | AATTAATATGCCAAAAATTGACGGATTATCGGTTGCAAAAAAG | ModR_D55E_ |
| ModR-D55E-R | TCCATTAAAACAATTTCAGGTTTAAACTCCTCAAATACTTTTAATGCC | ModR_D55E_ |
| ModR-D55A-F | CTATTAATATGCCAAAAATTGACGGATTATCGGTTGCAAAAAAG | ModR_D55A_ |
| ModR-D55A-R | CCATTAAAACAATTTCAGGTTTAAACTCCTCAAATACTTTTAATGCC | ModR_D55A_ |
| ModS_F | CTTGATGGAATGGACGATTGGG | qRT-PCR |
| ModS_R | TTGAAGATACTCATGAAGTGCTTGG | qRT-PCR |
| ModR_F | CTTCAACGGGAGAAGAGGCA | qRT-PCR |
| ModR_R | AGCCGTTTGTGCATAATCAAAAT | qRT-PCR |
| MsrAB_F | CTCAATGTGGATGGCCTAGTTT | qRT-PCR |
| MsrAB_R | AATGTGCTTTTCCACTTCTGCT | qRT-PCR |
| Trx_F | AGCTTTTGGAGCAGAGATGGA | qRT-PCR |
| Trx_R | GGACACCAAGAAGCCCAAAAC | qRT-PCR |
| CcdA_F | GGATGGACACCTTGTGTTGGA | qRT-PCR |
| CcdA_R | AAGGTGTCGCTAAACCTAAAACA | qRT-PCR |
| NhaC-F | GGGAAAGTGCAGCTATTCTTGA | qRT-PCR |
| NhaC-R | GGAGCAACTTTATCTCCAGCAA | qRT-PCR |
| NifJ_F | TGGTATGCATGTTGGAGTAGAAG | qRT-PCR |
| NifJ_R | TCACGGAATAAGCCCTGTAATG | qRT-PCR |
| PfkB_F | TACTGTTGGTGCAGGAGATTC | qRT-PCR |
| PfkB_R | CACAAGCCACTGCAAATCTAAA | qRT-PCR |
| PurE_F | CAGGACTTGCAGCACATTTAC | qRT-PCR |
| PurE_R | ACAGTGCATCTAAACCTTCCA | qRT-PCR |
| Fap2_F | CAAGGAACTGGAAATGGAATAATAGG | qRT-PCR |
| Fap2_R | GGAGAAGTAGCAGCAACAGTAT | qRT-PCR |
| RpoD_F | AGGGAACATAGGCCTTATGAAAG | qRT-PCR |
| RpoD_R | GCCTGTCTTATCCACCAAGTAG | qRT-PCR |
| RT-16S-F | GGTTAAGTCCCGCAACGA | qRT-PCR |
| RT-16S-R | CATCCCCACCTTCCTCCTAC | qRT-PCR |

^a^ Engineered restriction sites are underlined.
